# Aging Alters Daily and Regional Calretinin Neuronal Expression in the Rat Non-image Forming Visual Thalamus

**DOI:** 10.3389/fnagi.2021.613305

**Published:** 2021-02-24

**Authors:** Felipe P. Fiuza, José Pablo G. Queiroz, Antônio Carlos Q. Aquino, Diego A. Câmara, Luiz Eduardo M. Brandão, Ramon H. Lima, José Rodolfo L. P. Cavalcanti, Rovena Clara G. J. Engelberth, Jeferson S. Cavalcante

**Affiliations:** ^1^Graduate Program in Neuroengineering, Edmond and Lily Safra International Institute of Neuroscience, Santos Dumont Institute, Macaíba, Brazil; ^2^Laboratory of Neurochemical Studies, Department of Physiology, Biosciences Center, Federal University of Rio Grande do Norte, Natal, Brazil; ^3^Department of Medical Sciences, Uppsala University, Uppsala, Sweden; ^4^Laboratory of Experimental Neurology, Department of Biomedical Sciences, Health Science Center, University of State of Rio Grande do Norte, Mossoró, Brazil

**Keywords:** aging, intergeniculate leaflet, ventral lateral geniculate nucleus, lateral geniculate body, calcium binding proteins, circadian rhythms, calretinin, stereology

## Abstract

Aging affects the overall physiology, including the image-forming and non-image forming visual systems. Among the components of the latter, the thalamic retinorecipient inter-geniculate leaflet (IGL) and ventral lateral geniculate (vLGN) nucleus conveys light information to subcortical regions, adjusting visuomotor, and circadian functions. It is noteworthy that several visual related cells, such as neuronal subpopulations in the IGL and vLGN are neurochemically characterized by the presence of calcium binding proteins. Calretinin (CR), a representative of such proteins, denotes region-specificity in a temporal manner by variable day–night expression. In parallel, age-related brain dysfunction and neurodegeneration are associated with abnormal intracellular concentrations of calcium. Here, we investigated whether daily changes in the number of CR neurons are a feature of the aged IGL and vLGN in rats. To this end, we perfused rats, ranging from 3 to 24 months of age, within distinct phases of the day, namely zeitgeber times (ZTs). Then, we evaluated CR immunolabeling through design-based stereological cell estimation. We observed distinct daily rhythms of CR expression in the IGL and in both the retinorecipient (vLGNe) and non-retinorecipient (vLGNi) portions of the vLGN. In the ZT 6, the middle of the light phase, the CR cells are reduced with aging in the IGL and vLGNe. In the ZT 12, the transition between light to dark, an age-related CR loss was found in all nuclei. While CR expression predominates in specific spatial domains of vLGN, age-related changes appear not to be restricted at particular portions. No alterations were found in the dark/light transition or in the middle of the dark phase, ZTs 0, and 18, respectively. These results are relevant in the understanding of how aging shifts the phenotype of visual related cells at topographically organized channels of visuomotor and circadian processing.

## Introduction

Aging is characterized, for most living organisms, as a time-dependent physiological decline associated with increases in mortality and decreases in fertility rates (Flatt and Partridge, 2018). A practical approach to understand how the aging phenotype is determined, as well as the relationship between normal aging and age-related pathologies, is to identify distinctive cellular alterations as hallmarks of the aging process (López-Otín et al., 2013). In the nervous system, for instance, despite there being few changes in the global neuronal numbers throughout life (Long et al., 1999; von Bartheld et al., 2016), neurochemical-specific subpopulations of cells are lost during aging (Bañuelos et al., 2013; Pal et al., 2019; Lamerand et al., 2020). Thus, given the diverse nature of brain neurochemistry, the characterization of age-related changes in a cellular level still poses a challenging endeavor for neuroscience.

There is clear evidence that age-related brain dysfunction and neurodegeneration are associated with abnormal intracellular concentrations of calcium (Ca^2+^), as stated by the Ca^2+^ hypothesis of aging (Landfield, 1987; Khachaturian, 1994). Therefore, protein-mediated mechanisms of cytosolic Ca^2+^ buffering, such as the action of the EF-hand family of calcium binding proteins (CaBPs), may act as neuroprotective factors influencing age-related alterations (Alzheimer's Association Calcium Hypothesis Workgroup, 2017). Calretinin (CR), a representative of such CaBPs, seems to participate in the induction of long-term potentiation (Schurmans et al., 1997) and is present in neurons which are selectively resistant to the toxicity induced by β-amyloid protein, calcium overload, and excitatory amino acid stimulation in the brains of rodents (Lukas and Jones, 1994; Pike and Cotman, 1995). The CR and other CaBPs are often employed as histological markers due to their complementary distribution across brain regions and neuronal subclasses, being linked with region-specific vulnerability to aging effects (Fairless et al., 2019; Lamerand et al., 2020). Accordingly, the CR immunopositive (CR+) neurons are lost during aging in the cortical and subcortical regions of rodent and human brains (Villa et al., 1994; Bu et al., 2003; Bae et al., 2015; Ahn et al., 2017).

Aging affects the overall physiology, including visual, and circadian functions (Kondratova and Kondratov, 2012; Engelberth et al., 2013; Yan et al., 2020). After light reaches the retina, visual pathways to the brain nuclei are commonly grouped into image-forming and non-image forming visual systems (Lucas et al., 2014; Sondereker et al., 2020). A major retinorecipient zone lies in the thalamic lateral geniculate body, which is subdivided into dorsal lateral geniculate nucleus (dLGN), intergeniculate leaflet (IGL), and ventral lateral geniculate nucleus (vLGN) (Morin and Studholme, 2014). Apart from the clear subdivision of the vLGN in an external retinorecipient (vLGNe) and an internal non-retinorecipient (vLGNi) portions, there is no obvious cytoarchitectonic differences in these regions (Monavarfeshani et al., 2017). Unlike the dLGN, the classical component of the image forming visual system, the IGL and vLGN have no thalamocortical relays to any cortical areas (Harrington, 1997; Monavarfeshani et al., 2017). Instead, the IGL and vLGN widely innervate subcortical visuomotor related nuclei, such as pretectal and optic accessory system regions, being likely the main thalamic source of afferents to the superior colliculus in rats (Matute and Streit, 1985; Harrington, 1997). Also, the IGL is known to provide photic and non-photic (e.g., novel or metabolic conditions) inputs to the hypothalamic suprachiasmatic nucleus (SCN) that fine-tunes the circadian photo-entrainment (Morin, 2013). For all these reasons, the IGL and vLGN are classified as the thalamic components of the non-image forming visual system (Fox and Guido, 2011; Chengetanai et al., 2020).

It is noteworthy that several visual-related nervous cells, such as the retinal ganglion cells (RGCs), SCN, IGL, and vLGN neurons are neurochemically characterized by the presence of CR (Arai et al., 1992; Jeon and Jeon, 1998; Lee et al., 2010a,b; Lee et al., 2016; Moore, 2016). Interestingly, the day–night variations in the CR expression denote region-specificity (Campos et al., 2015a,b). For instance, the rat SCN presents a higher density of CR+ neurons in the light phase in comparison with the dark phase of the day (Moore, 2016). Notably, age-related alterations in the daily rhythmicity of other neurochemicals, such as serotonin, noradrenaline, dopamine, arginine vasopressin, and vasoactive intestinal polypeptide, are features of the brains of rodents and primates (Míguez et al., 1998; Cayetanot et al., 2005; Jagota and Kalyani, 2008). In fact, aging leads to a loss of rhythm in 90% of the hippocampal proteins that display circadian expression in mice (Adler et al., 2020). Thus, given the relationship among aging, calcium signaling, and circadian functions, one could hypothesize that daily changes in the CaBPs expression are markers of cellular aging and regional vulnerability. Here, we use unbiased stereology, the gold standard of cell quantification, to assess whether daily changes in the number of CR+ neurons occur in the IGL and vLGN of rats from different ages. In addition to presenting new data regarding this scarcely investigated issue, we discuss how the interpretation of morphometric data benefits from taking into consideration the chronobiologic context. Moreover, we present data supporting that a phase-dependent reduction in the amplitude of CR expression characterizes an age-related shift of neuronal phenotypes in the rat non-image forming visual thalamus.

## Materials and Methods

### Experimental Subjects

A total of 48 male Wistar rats, ranging from 3 to 24 months of age, were housed in cages at 22°C and 50% humidity in a 12:12 h light/dark cycle with food and water freely available. Animals were divided into four groups (*n* = 12 per group) regarding the photic condition, or zeitgeber, in which euthanasia was performed. By definition, the zeitgeber time (ZT) 12 corresponds to the lights-off hour in the animal housing facility. Specifically, animals were euthanized at ZT 0-dark/light transition, ZT 6-light phase, ZT 12-light/dark transition, and ZT 18- dark phase. All procedures were approved by local ethics committee (CEUA-UFRN number 054/2015) in accordance with Brazilian law number 11.794/2008 for animal experimentation.

### Tissue Fixation

Following anesthesia with sodium thiopental (40 mg/kg), animals were submitted for a thoracotomy and transcardiac perfusion through the left ventricle with a 300 ml NaCl solution (0.9%) followed by 300 ml formalin (10%) in a 0.1 m phosphate buffer (PB, pH 7.4). These procedures were carried out in the beginning of each ZT. Following perfusion, the brains were removed, post-fixed with the same fixative overnight and cryoprotected in a 30% sucrose solution with 0.1 m PB (pH 7. 4) for 3 days. Then, brains were sectioned into 50 μm coronal slices in a cryostat (Leica Microsystems) and the sections were sequentially collected in 96-well plates filled with antifreezing solution to be stored at −20°C until use for immunohistochemistry. We further detailed tissue sampling under the Stereology topic.

### Immunohistochemistry

Sections of IGL and vLGN were submitted to free-floating immunohistochemistry. Firstly, sections were blocked for endogenous peroxidase activity in 0.3% hydrogen peroxide. Then, sections were incubated overnight with rabbit anti-CR primary antibody (C7479, Sigma-Aldrich) in a 1:1,000 dilution with 2% bovine serum albumin and 0.1 m PB with 0.4% Triton X-100 (PBTX 0.4%). After rinsing, the sections were incubated with biotinylated goat anti-rabbit secondary antibody (111-065-003, Jackson ImmunoResearch) in a 1:1,000 dilution with PBTX 0.4%. Then, sections were incubated in a 0.5% avidin-biotin solution (Vectastain standard ABC kit, PK-4000, Vector Laboratories), with 2.3% NaCl addiction, for 120 min. Finally, sections were placed with a 2.5% solution of diaminobenzidine (DAB) diluted with 0.1 m PB. The final reaction was performed adding a 0.01% H_2_O_2_ solution, to reveal brown-stained areas resulting from DAB oxidation. All sections were simultaneously removed from the DAB solution after 2 min of reaction to minimize the staining variability. The brain sections were mounted on gelatinized slides, dried, dehydrated in graded ethanol solutions, cleared in xylene, and coverslipped with a DPX embedding matrix. Prior to histological processing, we performed a pilot study to establish optimal antibody concentration and incubation time. We confirmed internal positive controls by observing well-defined CR+ cell bodies in regions such as the cerebral cortex, the thalamic lateral posterior complex, and in the caudal zona incerta. Also, we addressed negative controls by incubating sections with no addition of primary antibody. In these cases, we observed no immunoreactivity.

### Stereology

Unbiased stereological analyses were performed in an BX61 optical microscope (Olympus, Japan) fitted with a ProScan II X-Y-Z motorized stage (Prior Scientific, Rockland, MA, United States), a 0.5 μm resolution Heidenhain MT12 microcator (Heidenhain, Traunreut, Germany) and a Olympus DP-71 digital camera connected to a computer running the software NewCAST (Visiopharm, Hørsholm, Denmark). A pilot study was performed to establish optimal parameters of section fraction, counting frame area, grid-spacing size, and disector height for stereological analyses. Accordingly, we used a 1/4 section sampling fraction (ssf), corresponding to ~5 IGL and 6 vLGN sections from an average of 24 sections identified with the aid of the seventh edition of the rat brain atlas in stereotaxic coordinates (Paxinos and Watson, 2014). We took into account, the anatomical considerations of Arai et al. (1992) and Harrington (1997), as well as previous observations of our group (Fiuza et al., 2016, 2017), to establish all stereological parameters and to trace the regional boundaries. Our systematic uniform random sampling was established by randomly choosing the first section and picking every following vLGN section in a 200 μm interval.

#### Regions of Interest

**Figure 1:**
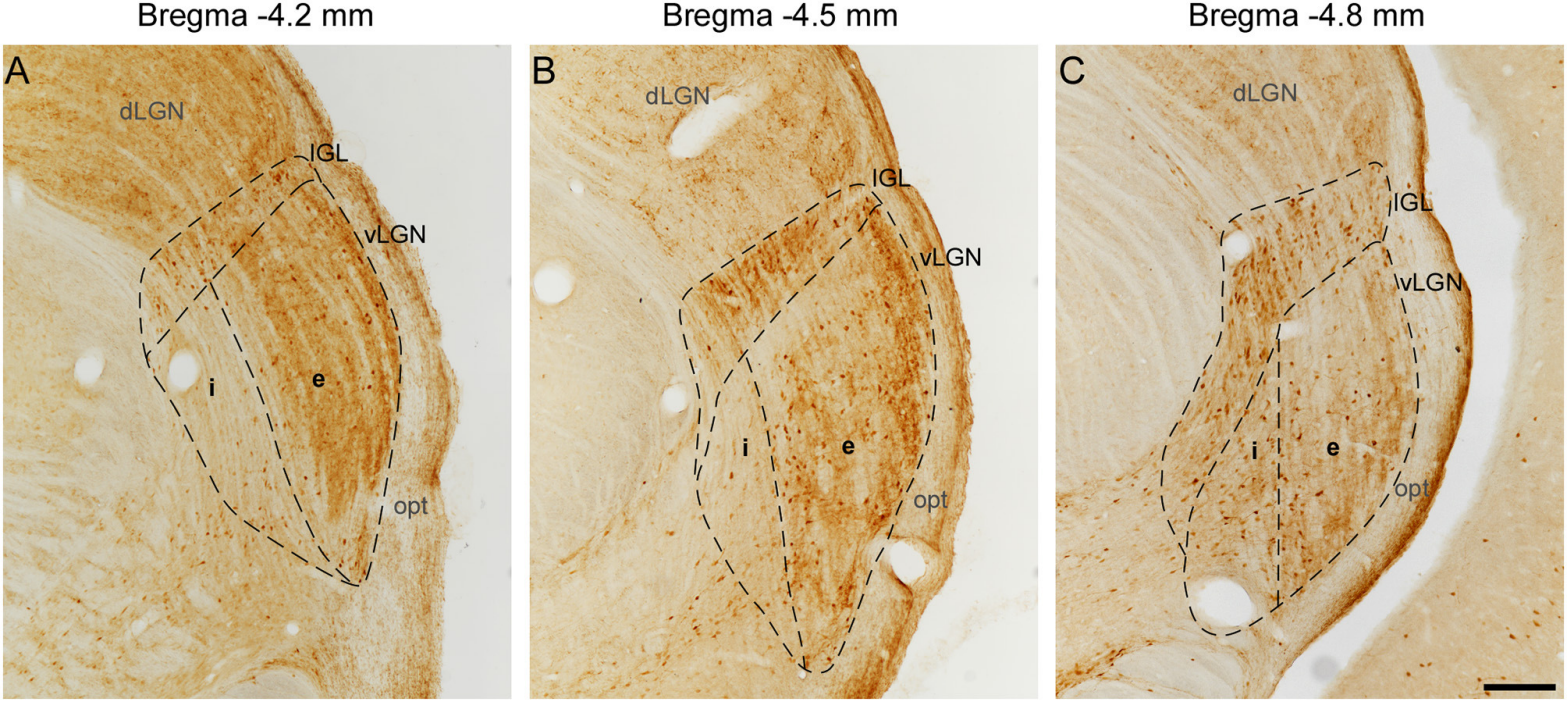
Delineation of IGL, vLGNe, and vLGNe in CR immunolabeled sections at distinct rostrocaudal positions. Distance caudal (–) to Bregma 0.0 (in mm) are indicated at the top of each panel. In the rat lateral geniculate body, dLGN, IGL, and vLGN are bordered laterally by the opt. **(A)** At −4.2 mm, vLGNi weak background aids in the delineation of its boundary with vLGNe. **(B,C)** At −4.5 and −4.8 mm, the medial portion of IGL projects ventrally and stronger immunoreactivity in CR cell bodies denoting the vLGNe/vLGNi border. Scale bar = 200 μm.

In the lateral geniculate body of the rat, the optic tract (opt) carries out retinal projections to the dLGN, IGL, and vLGN laterally bordering these nuclei (Figure 1). The dorsal and medial borders of IGL, namely the dLGN and superior thalamic radiation, were easily identified since there is little or no CR staining in its adjacent boundaries. As the dorsal most regions of the vLGNe and vLGNi are relatively spared from CR+ neurons, the IGL ventral borders can be distinguished from its adjacent vLGN nuclei. At rostral levels, the vLGNe presents a stronger background of CR staining which serves as a clear division from vLGNi (Figure 1A). At the middle and caudal levels, vLGNe enlarges and this stronger background is more pronounced in its surroundings, rather than in the center, still delineating the vLGNe/vLGNi border (Figures 1B,C). Also, the vLGNe neurons usually present a stronger CR cytoplasmic signal which aids in nuclei delineation.

#### Absolute Cell Number Estimation

We estimated absolute CR+ cell number bilaterally in an assumption-free manner for each nucleus following the optical fractionator method (West et al., 1991). Briefly, this methodology consists in overlaying a volumetric counting frame, the disector, in the histological material after the establishment of the ssf as well as area sampling fraction (asf) and height sampling fraction (hsf). The asf corresponds to the two-dimensional portion of the region of interest in which disectors are overlaid. The hsf comprehends the fraction of the Z plane that each disector probes. For our estimations, all samples were blind-coded so that the experimenter had no information regarding the age of the animal or its ZT group. First, we outlined the IGL and vLGN subdivisions with a low magnification objective (4x), and then counted the cells at high magnification (100x PlanApo oil lenses with 1.4 NA). We only counted the cells with the top of the nucleus clearly situated within the disector inclusion zone. Based on our pilot study, we determined a minimum of 100 counted cells to obtain reliable results with the coefficient of error (CE) below 0.1. To achieve this minimum cell number for counting, we used a 50 × 50 μm counting frame with a grid spacing size of 90 × 90 μm within our 1/4 ssf. We calculated the asf as a result of the counting frame area divided by grid-spacing area. The thickness of each post-shrinkage sections was calculated by focusing the topmost of tissue and slightly moving down to the bottommost focus point to measure the distance moved in the z-axis. As we observed that the tissue shrinks to an average value of 15 μm, the disector height was set at 12 μm with 1.5 μm guard zones above and below. Thus, the hsf was obtained through the division of the disector height by a number-weighted mean post-shrinkage section thickness. The CE was calculated with the smoothness class of m = 1 (Gundersen et al., 1999).

After obtaining cell counts (*Q*^−^) from IGL and vLGN, the total number of cells (*N*) was estimated by the optical fractionator equation (West et al., 1991):

$$\begin{array}{l} N=\sum Q^{-}.\frac{1}{ssf}.\frac{1}{asf}.\frac{1}{hsf} \end{array}$$

### Evaluation of CR Spatial Distribution

We followed the approach of Sabbagh et al. (2020) to evaluate whether CR staining predominates particular vLGN portions in the lateral to medial axis at different ZTs. As there was no indication for the existence of any kind of such cellular organization in the IGL (Monavarfeshani et al., 2017), we did not evaluate this nucleus. For this evaluation, we developed a custom-line scan script that runs in the public domain software ImageJ FIJI version 1.52p (National Institutes of Health, USA). First, the image is calibrated into μm scale and the user contours the region of interest. Then, the image is transformed into 8-bit type, subtracted from the background and inverted, displaying the CR staining as pixel gray values. Finally, equally spaced lines are overlaid into the region of interest and plot profiles show the variation of pixel intensity along each line length. The data are plotted based on the average intensity of all lines within the section and every ssf section of the nucleus. Considering the variable stoichiometry in immunohistochemical procedures (Bishop et al., 2018), comparing intensities between the age and ZT groups could retrieve biased results. For this reason, we did not perform any direct statistical comparison with this dataset. Instead, we used its descriptive feature to evaluate the relative spatial predominance of CR staining in animals at earlier or later points of the lifespan.

### Statistical Analysis

We performed a Kolmogorov–Smirnov test to assess the normality of our group distributions. After confirming that all distributions were normal, we computed Pearson's correlation coefficient and linear regression analysis to assess if there were any predictive relationships between age and CR cell numbers within each ZT group. Further, we divided our study population into three age groups, namely, young (3–7 months), middle-aged (12–18 months), and old (19–24 months) groups (Table 1). Then, we performed a two-way ANOVA followed by Tukey's test for *post-hoc* comparisons to compare the effects of age and ZT in CR cell numbers of each LGN nucleus. These analyses aided the identification of region-specific CR daily rhythmicity and to find whether the aging effects occurred in the earlier or later periods of animal lifespan. In all analyses, differences were considered significant at *p* ≤ 0.05 and data are expressed as mean ± SD. Data analyses were performed with the GraphPad prism version 7.0 software.

**Table 1:** Mean CR+ neuronal number estimated in the IGL, vLGNe, and vLGNi in each ZT and age groups.

**ZT group**	**Age group (Mean ± SD months)**	**Nucleus**		
		**IGL**	**vLGNe**	**vLGNi**
0	Young (4 ± 1.15)	1,889 ± 516	2,999 ± 459	1,916 ± 375
	Middle-aged (16 ± 2.70)	1,777 ± 525	2,634 ± 721	1,770 ± 472
	Old (23.25 ± 2.70)	1,950 ± 443	2,763 ± 180	1,713 ± 695
6	Young (4.5 ± 1.91)	2,533 ± 111	4,055 ± 619	2,040 ± 393
	Middle-aged (15 ± 2.16)	2,279 ± 421	3,088 ± 209^*^	2,129 ± 395
	Old (23.5 ± 0.58)	1,963 ± 240^*^	3,163 ± 218^*^	1,729 ± 430
12	Young (4.75 ± 1.70)	2,572 ± 232	3,480 ± 256	2,831 ± 148
	Middle-aged (15.25 ± 2.36)	2,397 ± 170	3,212 ± 155	2,415 ± 459
	Old (22 ± 1.41)	1,796 ± 87^*^^#^	2,754 ± 272^*^	2,101 ± 274^*^
18	Young (3.75 ± 0.95)	1,926 ± 442	2,909 ± 702	2,146 ± 312
	Middle-aged (14 ± 1.87)	1,874 ± 203	2,849 ± 316	1,901 ± 472
	Old (20 ± 1)	2,149 ± 50	3,393 ± 245	1,558 ± 162

* *p ≤ 0.05 in comparison with the young group within the same ZT group*.

# *p ≤ 0.05 in comparison with the middle-aged group within the same ZT group. Data are expressed as mean ± SD*.

*IGL, Intergeniculate leaflet; vLGNe, Ventral lateral geniculate nucleus external portion; vLGNi, Ventral lateral geniculate nucleus internal portion; ZT, Zeitgeber time*.

## Results

### Daily Rhythmicity of CR Expression

**Figure 2:**
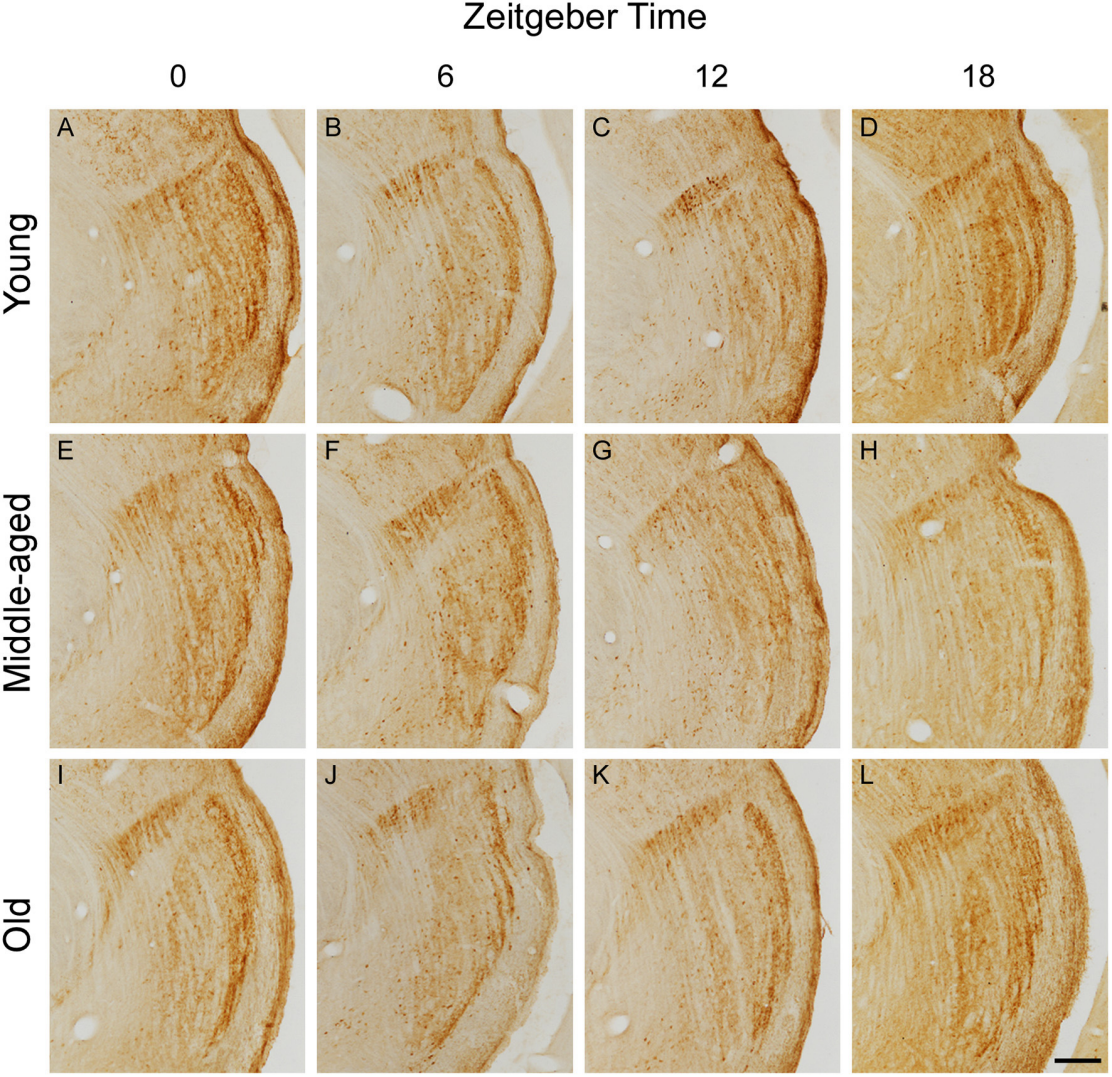
Photomicrographs of CR immunolabeled histological sections containing the IGL, vLGNe, and vLGNi in animals from distinct age and ZT groups. CR sections are obtained after brain perfusion at ZTs 0 (dark/light phase), 6 (light phase), 12 (light/dark phase), and 18 (dark phase) from young **(A–D)**, middle-aged **(E–H)**, or old **(I–L)** animals. Scale bar = 225 μm.

**Figure 3:**
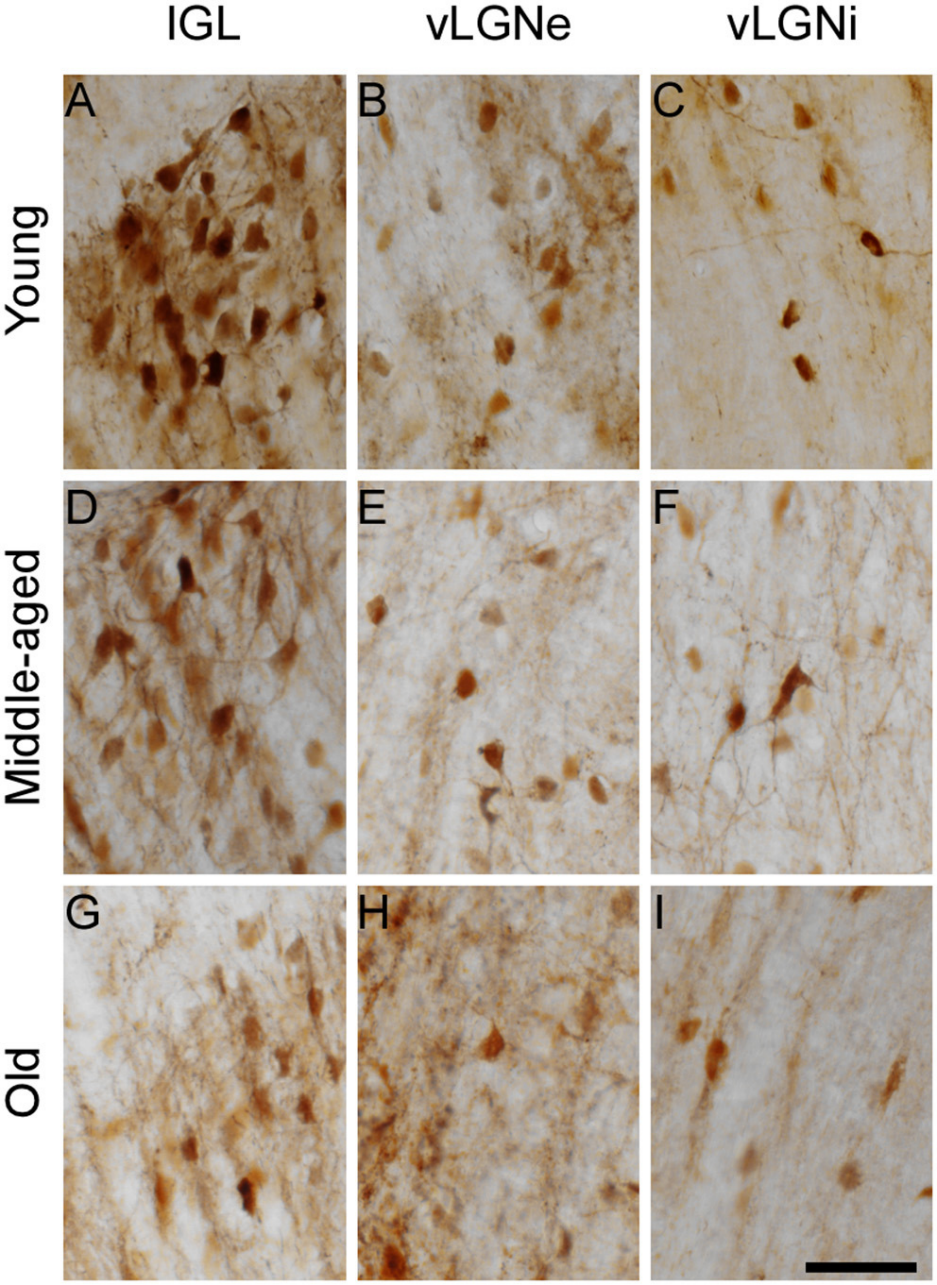
High-magnification photomicrographs of CR immunolabeled cells of the IGL, vLGNe, and vLGNi at ZT 12. The IGL, vLGNe, and vLGNiCR+ neurons from young **(A–C)**, middle-aged **(D–F)**, and old **(G–I)** animals are shown in detail. Scale bar = 50 μm.

**Figure 4:**
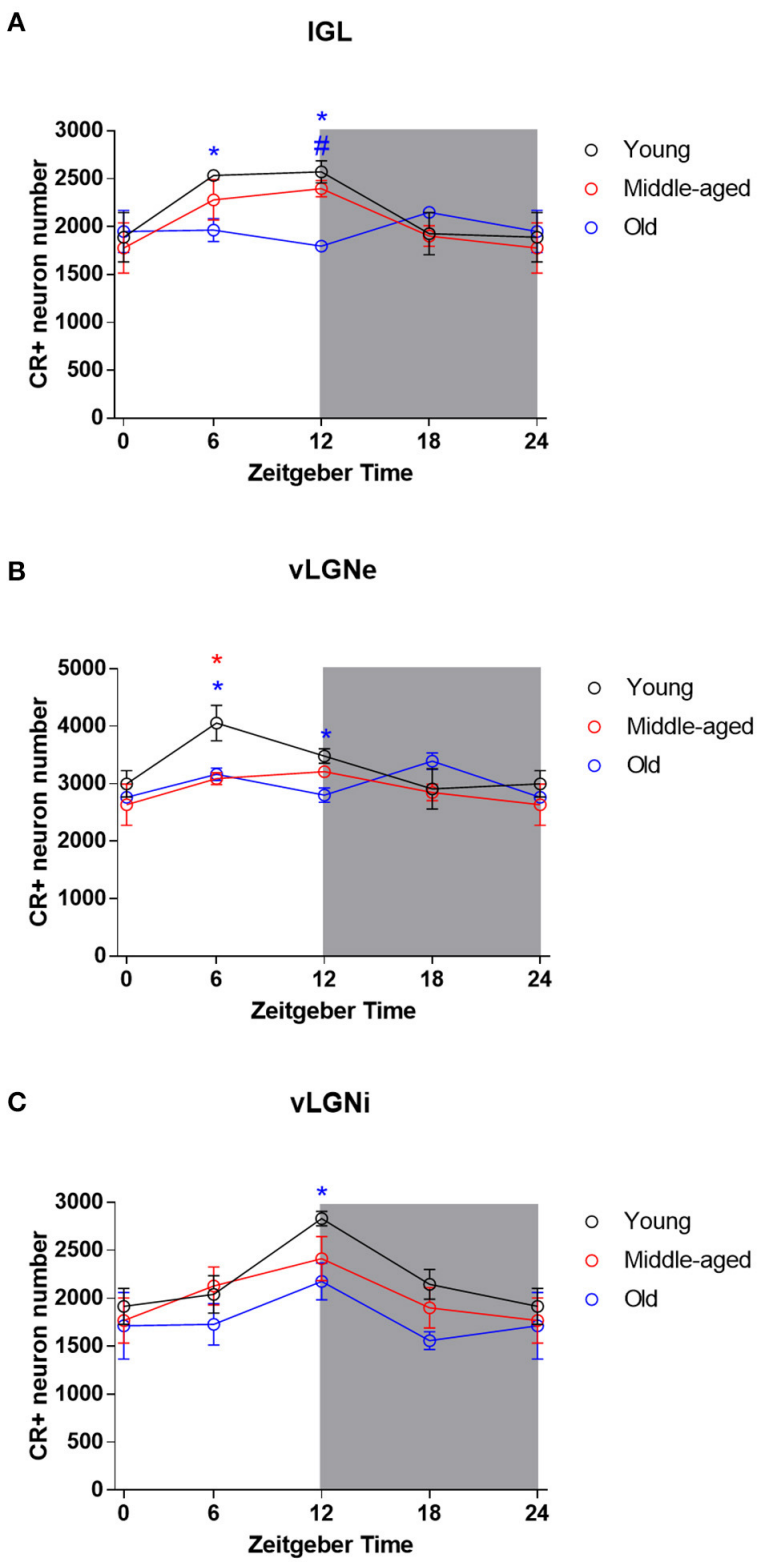
Daily rhythmicity of CR expression in the IGL, vLGNe, and vLGNi. Stereological estimations of total CR cell number at each ZT in the IGL **(A)**, vLGNe **(B)**, and vLGNi **(C)** from young (black), middle-aged (red), or old (blue) animals. Data from ZT 0 are double plotted as ZT 24, to graphically represent the 24 h of the day. Shaded gray areas represent the dark phase. Blue *p < 0.05 in young vs. old comparison. Blue #*p* < 0.05 in middle-aged vs. old comparison. Red *p < 0.05 in young vs. old comparison. Data are analyzed by two-way ANOVA followed by Tukey's test for *post-hoc* comparison. Data are plotted as mean ± SD.

Representative IGL and vLGN CR immunolabeled sections of each ZT group in rats from distinct ages are shown in Figure 2. In Figure 3, we show CR+ cells in higher magnification at ZT 12. After employing the two-way ANOVA with light condition as an isolated factor, we observed a significant effect upon CR neuronal changes in the IGL [F_(3,36)_ = 4.07; *p* = 0.01], vLGNe [F_(3,36)_ = 4.82; *p* = 0.006], and vLGNi [F_(3,36)_ = 5.99; *p* = 0.002]. In the IGL, Tukey's test for *post-hoc* comparisons revealed that young animals present a higher number of CR+ neurons at ZT 6 (2,533 ± 111), and ZT 12 (2,572 ± 232) in comparison with ZT 0 (1,889 ± 516). Also, we observed a significant reduction at the ZT 18 (1,926 ± 442) in comparison with ZT 12. In the vLGNe, Tukey's test for *post-hoc* comparisons revealed that young animals present a higher number of CR+ neurons at ZT 6 (4,055 ± 619) in comparison with ZT 0 (2,999 ± 459) and ZT 18 (2,909 ± 702). In the vLGNi, Tukey's test for *post-hoc* comparisons revealed that young animals present a higher number of CR+ neurons at the ZT 12 (2,831 ± 148) in comparison with ZT 0 (1,916 ± 375) and ZT 6 (2,040 ± 393). In middle-aged and old animals, we observed no alterations in CR numbers at different ZTs in any nucleus (Figure 4).

### Age-Related Changes in CR Cell Numbers of IGL and vLGN

Mean IGL, vLGNe, and vLGNi CR cell numbers of each age and ZT group are summarized in Table 1. In the IGL, two-way ANOVA with age as an isolated factor revealed no significant effects upon CR cell number alterations [F_(2,36)_ = 2.44; *p* = 0.10]. However, we found a significant effect between the interaction of age and ZT factors in the changes of CR cell numbers [F_(6,36)_ = 2.334; *p* = 0.05]. In the ZT 6, the Tukey's test for *post-hoc* comparisons revealed a significant CR cell number reduction in the old animals (1,963 ± 240) in comparison with the young ones (2,533 ± 111). In the ZT 12, we found a significant reduction of CR cells in old rats (1,796 ± 87) in comparison with both young (2,572 ± 232), and middle-aged (2,397 ± 170) groups (Figure 4A).

In the vLGNe, we observed CR cell numbers changed due to age factor [F_(2,36)_ = 4.6; *p* = 0.01] and the interaction between age and ZT factors [F_(6,36)_ = 2.51; *p* = 0.04]. In the ZT 6, Tukey's *post-hoc* test revealed a reduction in the CR cell numbers for the old (3,163 ± 218) and middle-aged (3,088 ± 209) groups in comparison with the young ones (4,055 ± 619). In the ZT 12, Tukey's *post-hoc* test revealed a significant reduction in CR cell numbers in the old (2,754 ± 272) animals in comparison with the young (3,480 ± 256) group (Figure 4B).

In the vLGNi, we observed CR cell numbers changed due to age factor [F_(2,36)_ = 4.73; *p* = 0.01] but the interaction between age and ZT factors had no significant effects [F_(6,36)_ = 0.48; *p* = 0.8]. In the ZT 12, Tukey's *post-hoc* test revealed a reduction in CR cell numbers of old (2,101 ± 274) rats in comparison with the young ones (2,831 ± 148) (Figure 4C).

**Figure 5:**
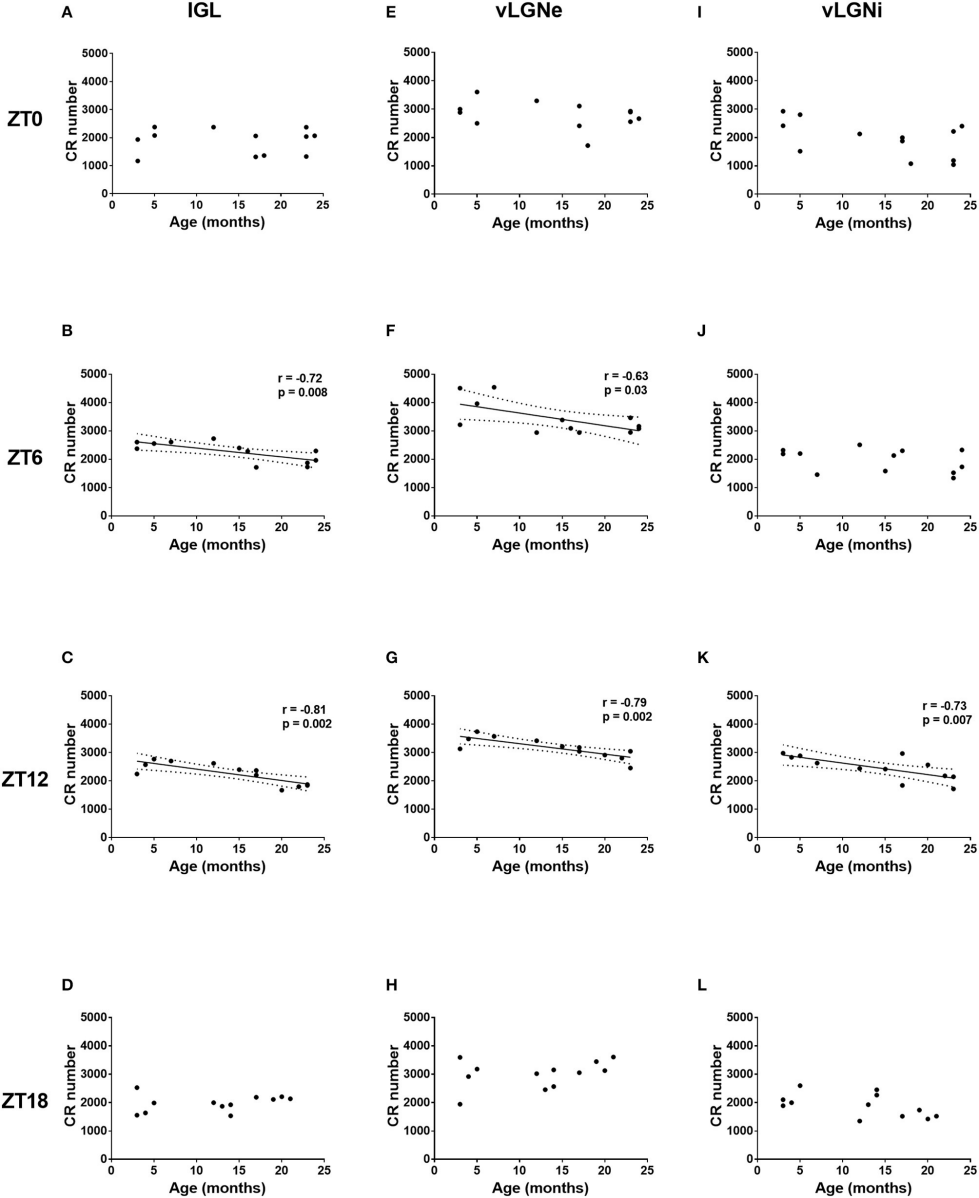
Age-related changes in the CR expression of the IGL, vLGNe, and vLGNi. Total CR+ cell numbers of the IGL **(A–D)**, vLGNe **(E–H)**, and vLGNi **(I–L)** are plotted as a function of age in each ZT. Pearson's correlation coefficient (*r*), regression lines with 95% confidence intervals and *p-*values are shown when a significant linear relation is observed.

Considering age as a continuous variable, we reported negative correlations between age and CR cell numbers in IGL (*r* = –0.72, *p* = 0.008) and vLGNe (*r* = –0.63, *p* = 0.03) at the ZT 6. Also, we found negative correlations between age and CR cell numbers in IGL (*r* = −0.81, *p* = 0.002), vLGNe (*r* = –0.79, *p* = 0.002), and vLGNi at the ZT 12 (*r* = –0.73, *p* = 0.007). We observed no correlations between these two variables in any nucleus in the other ZT groups (Figure 5).

### Spatial Distribution of CR in the vLGN

**Figure 6:**
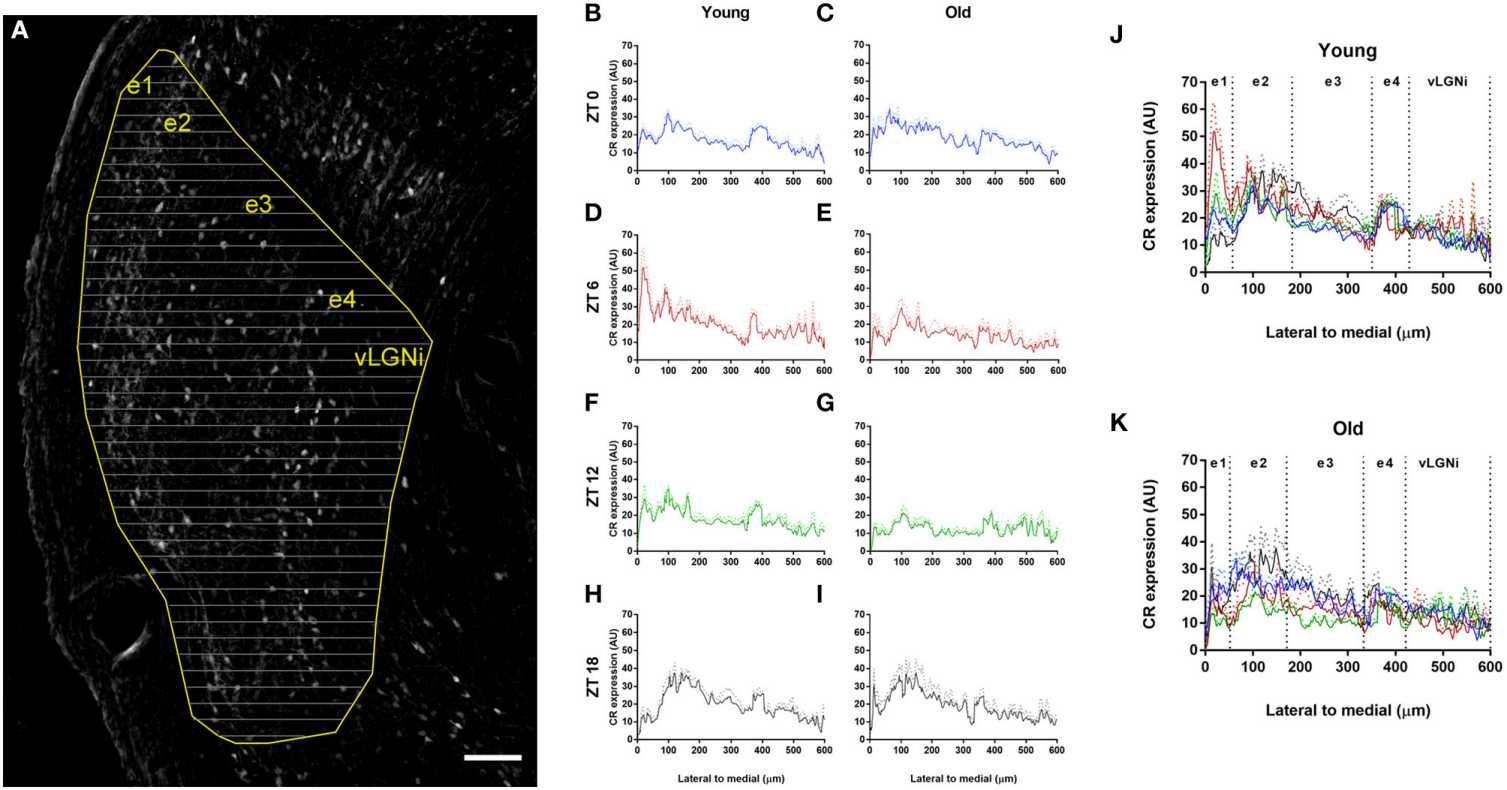
Spatial distribution of CR staining in the vLGN. **(A)** Photomicrograph of the vLGN digitally processed for background subtraction. The vLGN borders are drawn (yellow) and equally spaced lines (gray) in the lateral to medial axis are overlaid to plot profiles of pixel intensity variations in arbitrary units (AU). Scale bar = 100 μm. **(B–I)** CR staining levels through the lateral to medial axis in the young and old animals at t ZTs 0 (blue), 6 (red), 12 (green), and 18 (black). **(J,K)** Every ZT plot overlaid for the young and old animals. Relative CR predominance distinguishes five zones, namely, e1, e2, e3, e4, and vLGNi domains. Data are plotted as mean gray values with standard error for each group.

Through line scan analysis, we distinguished four spatial vLGNe and one vLGNi domains marked by CR staining (Figure 6). We refer to those as e1, e2, e3, e4, and vLGNi zones. As different animals have slight differences in vLGN length, here we use approximations of average distances we found for the young group. From the lateral-most border to 50 μm, we observed that the e1 zone is relatively spared from CR (young: 16.34 ± 4.40; old: 14.36 ± 4.05). In young animals at the ZT 6, however, we found a higher predominance of CR staining in this area (35.17 ± 11.11). Between e1 and 190 μm, we observed the e2 CR rich area (young: 24.74 ± 5.15; old: 21.55 ± 3.69). Between e2 and 350 μm, we found another CR-scarce zone that we refer as e3 (young: 16.54 ± 1.98; old: 14.93 ± 2.47). Between e3 and 420 μm, we report the e4 CR rich area highlighting the medial-most vLGNe border with vLGNi (young: 20.49 ± 4.36; old: 17.09 ± 4.04). From e4 to the medial-most boundary of vLGNi, we observed no particular pattern of CR staining (young: 11.62 ± 4.48; old: 10.20 ± 3.84). Data for CR intensity in each age group are presented here as the average of all ZTs, except for the e1 in which ZT 6 was isolated.

## Discussion

In this study, we describe how changes in the daily expression of the calcium binding protein, CR, marks region-specific patterns of cellular aging in the rat non-image forming visual thalamus. In a previous work, we quantified the total neuronal number for each of the subregions analyzed here using the same age-groups (Fiuza et al., 2017). Comparing these datasets, we can infer CR+ neurons correspond to 32–45% of the total neuronal population in the IGL, 16–23% in the vLGNe, and 13–20% in the vLGNi of rats. Considering CR is a known marker for GABAergic interneurons in other regions such as the cerebral cortex and amygdaloid complex (Tremblay et al., 2016; García-Amado and Prensa, 2021), it is likely that a sizeable portion of these neurons also composes GABAergic neuronal subpopulations. In fact, almost the entirety, if not all, of IGL cells are immunopositive for GAD, an enzyme that synthesizes GABA (Moore and Speh, 1993; Langel et al., 2018). Also, Gad1 and Gad2 mRNA, which translates the GAD 67 and GAD 65 isoforms, are expressed by 25 and 41%, respectively, of the total DAPI stained cells in the mouse vLGN (Sabbagh et al., 2020). It is important to highlight that CR+ neurons are also present in the geniculate body of lizard (Dávila et al., 2000), African wild dog (Chengetanai et al., 2020), banded mongoose, ferret (Pillay et al., 2020), marmoset, rock cavy (Cavalcante et al., 2008), and human brains (Münkle et al., 2000) denoting this characteristic is conserved, at some extent, during evolution. Since the IGL and vLGN contribute to visuomotor and circadian functions through projections to the superior colliculus, the pretectal nuclei, and suprachiasmatic nucleus in many species (Harrington, 1997; Morin, 2013), our findings could bring an interesting perspective for further translational aging studies.

We observed a phase-dependent CR expression in the IGL, vLGNe, and vLGNi of young rats. While every nucleus presented a higher number of CR cells during the light phase of the day, distinct phase-dependent patterns denoted a regional variability. In the IGL, we estimated higher numbers of CR+ cells in both ZTs 6 and 12 in comparison with ZT 18. However, CR+ cells peak only at ZT 6 in vLGNe and only at ZT 12 in vLGNi. Such a daily rhythmicity of CR expression, with higher CR+ counts at ZT 6 in comparison with ZTs 14 and 19, is also a feature of a neuronal subpopulation found in the rat suprachiasmatic nucleus (Moore, 2016). Additionally, in *Sapajus apella*, a diurnal primate, a similar pattern of reduced CR expression in the dark phase occurs in the ventral and dorsal subdivisions of the medial geniculate body and in the granular cell layer, but not in the polymorphic layer, of the dentate gyrus (Campos et al., 2015a,b). It is also noteworthy that, contrary to what we found for CR, no daily rhythms were detected for the CaBP calbindin D-28k (CB) in the adult rat IGL (Arvanitogiannis et al., 2000). Therefore, it is conceivable that CB and CR characterize distinct neuronal subpopulations in the IGL as occurs in the rat arcuate nucleus and in the human temporal cortex (del Río and DeFelipe, 1996; Foo et al., 2014).

Although daily rhythms of CR expression might reflect important intracellular mechanisms of calcium buffering and signaling, we showed here that it progressively declines with age in the rat non-image forming thalamus. In fact, middle-aged and old animals were completely absent of daily variations in the CR+ cell number of every nucleus. To the best of our knowledge, only one other study addressed the question of whether daily-rhythms of CaBP expression could be a hallmark of cellular aging. In this study, no daily or age-related changes in the density of CB immunopositive neurons were observed in the SCN of the primate, *Microcebus murinus* (Cayetanot et al., 2007). Here, we highlight time of the day as an important methodological consideration for CaBP cell number estimation as two independent studies carried out at isolated ZTs, for instance, one at ZT 12 and other at ZT18, could report either age-related neuronal loss or no alterations whatsoever.

Treating age as a continuous variable, we observed age-related reduction in CR+ neurons in all three nuclei at the ZT 12. At ZT 6, we found such alterations only in IGL and vLGNe. No changes were found in any nucleus at the other ZTs. On comparing with the age categories dataset, such neuronal loss seems to occur at different rates. At the ZT 6 we can detect changes in CR+ neurons of vLGNe in middle-aged rats whereas in the other nuclei at ZT 6 and in every nucleus at ZT 12, the cell numbers were altered only in the old animals. Considering the total neuronal number of these regions remains stable during aging (Fiuza et al., 2017), our findings here are likely due to loss in the capacity of neurons to express CR rather than death of CR+ neuronal population with aging. Such findings add up to a number of reports regarding age-related reductions in CR+ neurons in the hippocampus (Villa et al., 1994), inferior colliculus (Ouda et al., 2012), striatum (Bae et al., 2015), and somatosensory cortex (Ahn et al., 2017) of rodent brains, as well as in the temporal and auditory regions of the human cerebral cortex (Bu et al., 2003). Age-related pathologies also result in decreased number of CR+ neuronal population, as observed in the olfactory cortex of transgenic AβPP/PS1 mice (Saiz-Sanchez et al., 2012) and in the pyriform cortex of patients with Alzheimer's disease (Saiz-Sanchez et al., 2015). In some scenarios, these alterations might be due to a specific reduction of CR expression rather than cell death as we observed here. For instance, the number of CR+ large striatal interneurons, but not the absolute number of these cells, is decreased in Huntington's disease (Massouh et al., 2008). Notwithstanding, in the rat dorsomedial and ventromedial hypothalamic nuclei the percentage of CR+ neurons increases during aging (Vishnyakova et al., 2020). This reinforces the usefulness in understanding CR, or any CaBP, expression as regional markers of cellular changes in normal aging and in the progression of age-related diseases.

Through our analysis of spatial distribution, we observed a predominance of CR staining into the lateral-most and medial-most portions of the vLGNe, with a low density CR zone between these regions, at all ZTs. In the vLGNi, however, we distinguished no specific pattern of CR distribution. That was the case even in the old animals, suggesting that the age-related cell loss we observed is not restricted at a particular subset of spatially-located neurons, except perhaps in the e1 subdivision at ZT 6. We show that CR fairly delimits the vLGNe since sublaminae-specific markers reveal four hidden laminae, all containing GABAergic cells, in a lateral to medial organization (Sabbagh et al., 2020). It is noteworthy that in the vLGNe, but not in the IGL, retinal terminals are segregated into non-overlapping eye domains (Monavarfeshani et al., 2017). Such retinal projections to these nuclei arise from non-image forming RGCs, such as the M1 intrinsically photosensitive and Cdh3-GFP RGCs (Hattar et al., 2006; Osterhout et al., 2011). The lateral-most regions of the vLGNe, which we relate with CR delimited e1, presents retinal terminals from on–off direction-selective RGCs (Monavarfeshani et al., 2017). Also, rods and cones seem to participate in the IGL/vLGN activation since light-induced FOS immunoreactivity is reduced in these nuclei of mice lacking these photoreceptors, a response even more dampened in the aged animals (Lupi et al., 2012). Considering CR+ neurons in the IGL/vLGN may be targets from CR expressing RGCs (Arai et al., 1992), further works are necessary to establish whether a coincident loss of CR expression denotes age-related effects on topographically organized channels of visuomotor and circadian processing.

Unbiased stereological methods for quantitative neuroanatomy greatly enriched the knowledge of how aging affects the nervous system by directing a region-specific approach to detect age-related alterations in the cellular composition of the brain. As many studies focus in total protein quantification to address age-related changes, we highlight the informative feature to quantify cell numbers for an integrative approach. For instance, in here and in a few previous reports, if any, the CR+ cell bodies were found in the rodent dLGN (Arai et al., 1992; Evangelio et al., 2018). However, in comparison with IGL and vLGN, the mouse dLGN presents similar CR levels detected by Western blot and even higher expression of its associated Calb 2 mRNA (Sabbagh et al., 2018). While mRNA levels are not necessarily correlated with protein amounts (Maier et al., 2009), such findings might be due to the presence of CR in axon terminals at the dLGN as it occurs in the macaque prefrontal cortex (Melchitzky et al., 2005). Thus, mRNA and total protein expression approaches could benefit from the understanding if potential changes in molecule amount are accompanied by shifts in the cellular phenotype.

## Data Availability Statement

The raw data supporting the conclusions of this article will be made available by the authors, without undue reservation.

## Ethics Statement

The animal study was reviewed and approved by Comissão de ética no uso de animais (CEUA/UFRN).

## Author Contributions

FF, RE, and JC designed the study and wrote the paper receiving inputs from all the authors. FF, AA, DC, LB, and JC carried out histological procedures. FF, JQ, and RL performed image analysis and prepared figures. All authors contributed to the article and approved the submitted version.

## Conflict of Interest

The authors declare that the research was conducted in the absence of any commercial or financial relationships that could be construed as a potential conflict of interest.

## Abbreviations

asf: Area sampling fraction
CaBP: Calcium binding proteins
CB: Calbindin d-28k
CE: Coefficient of error
CR: Calretinin
CR+: Calretinin immunopositive
dLGN: Dorsal lateral geniculate nucleus
hsf: Height sampling fraction
IGL: Intergeniculate leaflet
opt: Optic tract
RGC: Retinal ganglion cell
SCN: Suprachiasmatic nucleus
ssf: Serial sampling fraction
vLGN: Ventral lateral geniculate nucleus
vLGNe: Ventral lateral geniculate nucleus external portion
vLGNi: Ventral lateral geniculate nucleus internal portion
ZT: Zeitgeber time
AU: Arbitrary units.
